# High Contrast Markings Can Negate the Benefits of Transparent Camouflage

**DOI:** 10.1002/ece3.73490

**Published:** 2026-04-10

**Authors:** Justin Yeager, Abigail Robison, Cordon D. Wade, James B. Barnett

**Affiliations:** ^1^ Grupo de Investigación en Biodiversidad, Medio Ambiente y Salud (BIOMAS), Facultad de Ingenierías y Ciencas Aplicadas Universidad de Las Américas Quito Ecuador; ^2^ Dirección General de Investigación y Vinculación Universidad de las Américas Quito Ecuador; ^3^ School of Natural Sciences Trinity College Dublin Dublin Ireland

**Keywords:** camouflage, Centrolenidae, crypsis, differential blending, disruptive coloration, glass frogs, silhouette, transparency

## Abstract

Transparency is, in theory, the ultimate form of concealment allowing for perfect background matching camouflage regardless of the environment. In nature, despite some remarkable examples of highly transparent organisms, physiological constraints mean that transparency is often partial or imperfect. This raises the question of how deviation from true transparency may affect detectability and how camouflage functions. Indeed, it has recently been suggested that partial transparency may function as disruptive camouflage as adjacent transparent and opaque patches differentially blend into the background. Differential blending may therefore offer a route by which obligate opaque structures may be concealed. The glass frogs (Centrolenidae) are a classic example of transparency with ventral skin that allows for a view of the internal organs. However, although the ventral skin is transparent and the frogs appear translucent, the internal organs are still largely opaque. Here we performed visual modelling and a field predation study with model frogs to ask how the degree of translucency and the arrangement of opaque structures affects detectability and survival. We predicted that greater translucency would improve concealment by facilitating more effective differential blending and that opaque elements which highlighted the recognisable shape of the frog would suffer increased predation risk. We found that greater translucency did improve background colour matching, but the presence of salient opaque patterns negated this camouflaging effect. However, the effect of salient patterning differed depending on model opacity, with outer markings receiving fewer attacks than central stripes for opaque but not translucent models. Here, survival was best explained by the distance at which the opaque patterning could be resolved, with thinner outer stripes receiving fewer attacks than thicker central stripes. Our data suggest that transparency may facilitate effective differential blending, but the efficacy of camouflage may be reduced by the presence of obligate opaque structures. These limitations to the efficacy of transparent camouflage may favour the evolution of translucency and explain why, despite having transparent ventral skin, glass frogs retain sparse green pigmentation in their dorsal skin.

## Graphical abstract

Transparency is perhaps theoretically the most efficient means of achieving camouflage; however, in application, species show considerable variance in their degree of transparency. We demonstrate the fragility of transparent camouflage, detailing how imperfections reduce efficacy with implications in predation risk.

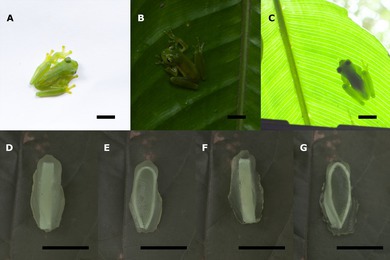

## 1 Introduction

Camouflaged animals use colours and patterns to blend into their surroundings and conceal their presence from their predators and/or prey (Cuthill 2019). Camouflage often relies on opaque pigments or structural colours on the animal's body which replicate or approximate the colours and patterns found in the environment (Cuthill et al. 2017; Cuthill 2019). However, being completely transparent offers animals an alternative where the environment itself is visible through their bodies (Cuthill et al. 2017; Barnett et al. 2025). Complete transparency therefore represents an idealised form of concealment where an organism is able to perfectly match its immediate surroundings in any environment (Johnsen 2001; Bagge 2019; Barnett et al. 2025).

**FIGURE 1:**
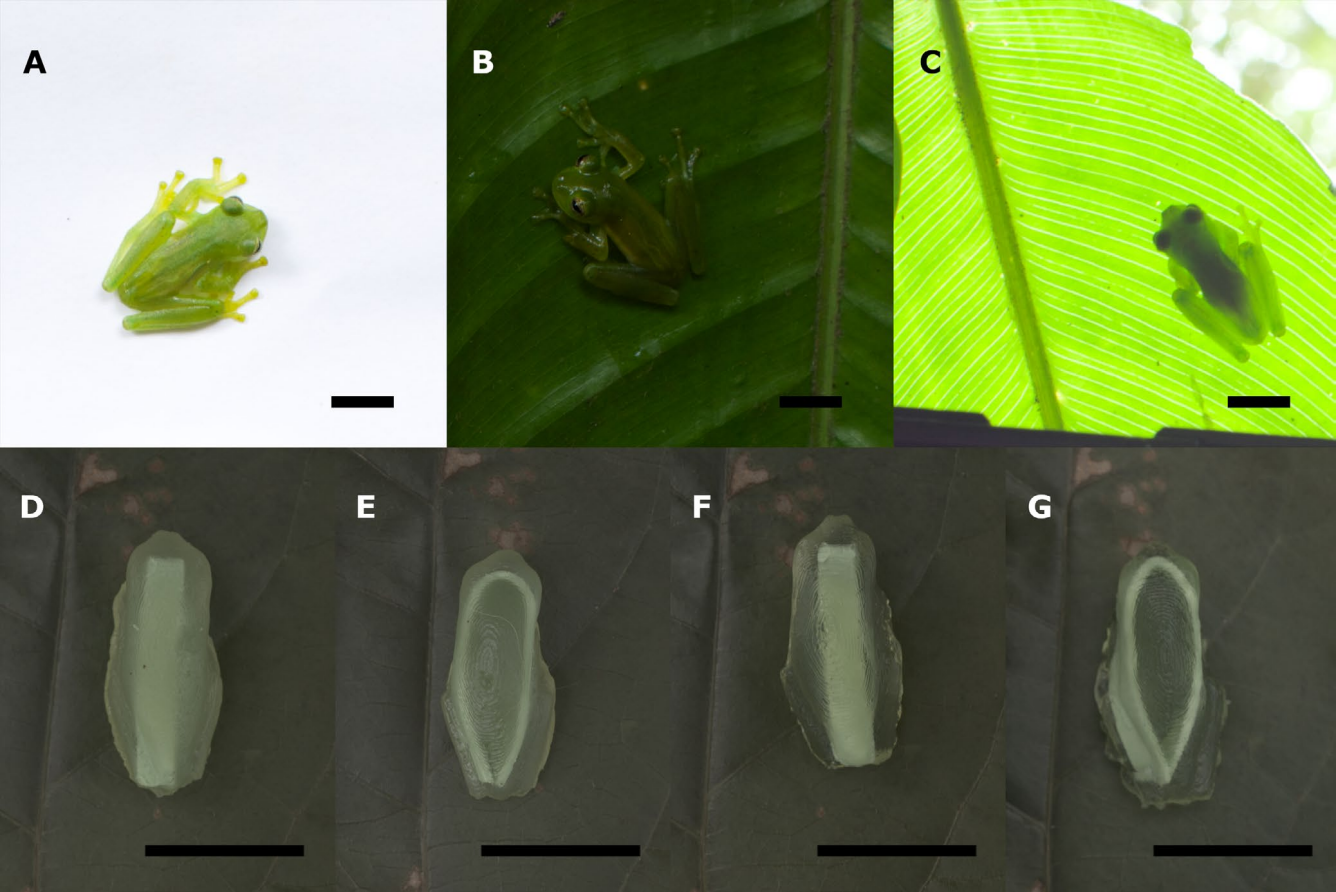
Examples of glass frog translucency and the four experimental treatments used in the predation study (scale bar = 10 mm). Top (A–C) *Espadarana prosoblepon* (a weakly transparent species) photographed (A) from above on a white background and (B, C) on a dark green leaf when viewed from above (B, with reflected light) and from below (C, with transmitted light). All photographs show the same individual (non‐linear images, photographed by Jim Barnett, 2019. Mindo, Ecuador). (A, B) The brightness of the dorsal green colouring appears to change depending on the background. In (B), the opaque parietal peritoneum covering the internal organs appears white and can be seen through the transparent lateral skin of the frog. (C) When backlit, the opaque eyes and internal organs cast a strong silhouette while the more translucent legs, and the posterior region of the body, better match the leaf. Bottom (D–G). The four experimental treatments used in the predation study. From left to right: D = OC, opaque‐centre, E = OE, opaque‐outline, F = TC, translucent‐centre and G = TE, translucent‐outline. All models were photographed on the same leaf (linear images, photographed by Leonardo Ávila, 2025. Quito, Ecuador).

The glass frogs (Centrolenidae) are a classic example of animal transparency with ventral skin which is often either partially or totally transparent, permitting the viewing of the internal organs. However, despite a lot of interspecific variation in transparency, the dorsal and lateral surfaces of glass frogs are predominantly green and pigmented (Guayasamin et al. 2020; Figure 1A,B). This means that, when viewed under natural conditions, rather than being fully transparent, the more ‘transparent’ species instead appear translucent (Barnett et al. 2020; Taboada et al. 2022), whereas others are nearly entirely opaque (Guayasamin et al. 2020). Although glass frogs are not entirely see‐through, translucency does still enhance camouflage by allowing the apparent brightness of the frog to shift towards that of its immediate background (Barnett et al. 2020). As these frogs largely sit on green leaves, this change in luminance allows their green colouring to darken or lighten to better match the green of the substrate directly beneath them (Barnett et al. 2020; Figure 1A,B).

To be transparent requires the modification of bodily tissues to minimise the absorption, scattering and reflectance of light across the whole visual spectrum (Johnsen 2001; Bagge 2019; Barnett et al. 2025). Therefore, while true transparency may be superior even to highly precise opaque background matching, deviations from perfect transparency, as seen in glass frogs, are common across a diverse set of species (Johnsen 2001; Bagge 2019; Gomez et al. 2021; Barnett et al. 2025). Deviations from complete transparency can include translucency (permitting diffuse light transmission but not a clear view through the organism), the presence of small, highly transparent, patches on otherwise opaque bodies (such as the clear ‘windows’ on the wings of many butterflies and moths), the presence of opaque structures within an otherwise transparent body (such as the eyes, internal organs, or bones), or conspicuous patches of opaque colour on an otherwise transparent or translucent body (Johnsen 2001; Bagge 2019; Gomez et al. 2021; Barnett et al. 2025). Such ‘imperfect’, or ‘partial’ transparency, may arise due to physiological constraints which compromise camouflage (Bagge 2019; Galloway et al. 2020) or as a hybrid strategy combining camouflage with other defensive or communicative signals (Arias et al. 2019; McClure et al. 2019; Yeager et al. 2024; Barnett et al. 2025). Glass frogs appear to address some of these constraints, and increase the degree of light transmission, by storing their red blood cells within their livers while at rest and by packaging their internal organs within a reflective sac that attenuates much of the incoming light (Taboada et al. 2022). Such adaptations suggest that these frogs may be under strong selection to evolve mechanisms which increase transparency. Yet, these structures still block and reflect a significant proportion of the incoming light and are only weakly translucent (Guayasamin et al. 2020; Taboada et al. 2022). The presence of seemingly obligate opaque structures, therefore, raises the question of how opaque patches may affect the efficacy of translucent camouflage in glass frogs as well as in many other transparent or translucent organisms.

One potential explanation is that incorporating some degree of transparency into opaque colour patterns can provide disruptive camouflage (Stevens and Merilaita 2008; Merilaita et al. 2017; Barnett et al. 2025). Recent research on transparent winged butterflies and moths, for example, suggests that small adjacent patches of opacity and transparency can blend into different features within the background to different degrees. This ‘differential blending’ can then break up the outline and the surface continuity of the organism into a series of unrecognisable features (Arias et al. 2020, 2021; Costello et al. 2020). Indeed, it has also been suggested that incorporating transparency into camouflage may disguise the size and shape of the silhouette (Barnett et al. 2020, 2025; Johnsen 2014; Rydell et al. 2020; Figure 1C). Little is known about the diurnal resting behaviour and predators of glass frogs, however this ‘silhouette concealment’ may allow disruptive camouflage to continue functioning even when an organism is backlit against a translucent substrate such as a leaf. This hypothesis has not yet been studied in detail (Barnett et al. 2025), and explicit evidence of differential predation based on perch height is currently unavailable.

Moreover, combinations of transparency and pigmentation may also integrate multiple functions simultaneously (Kikuchi et al. 2023; Barnett et al. 2025), with recent research on clearwing butterflies (Nymphalidae) suggesting that partial transparency or translucency can combine camouflage with conspicuous signals such as aposematism (Arias et al. 2019; McClure et al. 2019; Yeager et al. 2024). However, while it has been speculated that the addition of even small transparent patches contributes to camouflage in some meaningful way, just how it could work mechanistically remains unresolved.

Here, we used glass frogs as a model system to explore how the degree of transparency, and the arrangement of conspicuous, opaque, elements, may affect the efficacy of camouflage. We presented gelatine model frogs, with translucent or opaque bodies and two different opaque patterns (reminiscent of the opaque spine, pelvic girdle, and/or viscera which can be visible through the dorsal skin of certain glass frog species; Guayasamin et al. 2020), to wild birds and recorded the rate at which our models were predated (Figure 1). We hypothesised that greater translucency, and less recognisable opaque patterning (i.e., internal patterns which did not highlight the frog shape), would increase the degree of differential blending and decrease detectability. We therefore predicted that predation rates would be higher for opaque models and for those with opaque patterns which highlighted the recognisable outline of the frog. Moreover, as translucency could be especially beneficial in hiding the silhouette, we additionally varied model height. We predicted that any differences between treatments would be exaggerated for higher placed models as potential predators could approach from any direction and would therefore be more likely to approach from below where the silhouette would be visible. We then used visual modelling from calibrated photographs to assess how background colour and pattern matching was affected by the translucency of the gelatine and the arrangement of the clay, and to help elucidate the relationship between survival and detectability. Here we calculated how well the gelatine base, clay stripes and the mean colour of the frog models matched the colour and patterning of the leaf background as viewed by potential predators.

## 2 Methods

To examine how differences in translucency and in opaque patterning may affect camouflage we created artificial glass frog‐like models and recorded the rate at which wild avian predators attacked these models in the field. These models followed methods outlined in Barnett et al. (2020) and were made of a translucent gelatine body containing patterns of opaque modelling clay. This approach approximates the background‐dependent change in apparent brightness observed in glass frogs such as *Teratohyla midas* and *Espadarana prosoblepon* (Barnett et al. 2020; Figure 1). We performed our predation experiment at a rainforest site in the Mashpi Reserve (Pichincha, Ecuador) where several sympatric species of glass frogs, which differ in their degree of translucency, are commonly found in microsympatry (M. Roldan pers. comm., JY pers. obvs.). To help explain any differences in predation rate, we then used visual modelling from calibrated UV–VIS photographs to quantify differences in the detectability of each component of our models as viewed by their avian predators.

### 2.1 Model Construction

We designed four model types representing all combinations of two levels of transparency (O = opaque; T = translucent) and two different opaque patterns (C = centre, a central dorsal stripe; E = outline, a dorsolateral ring around the snout and along each side of the body): OC = opaque with an opaque central stripe, OE = opaque with an opaque outline, TC = translucent with an opaque central stripe and TE = translucent with an opaque outline (Figure 1D–G). These treatments were designed to test fundamental questions about how translucent camouflage functions rather than to mimic any particular species of glass frog. However, central patterns approximated the placement of the opaque spine and viscera and did not highlight the outline of the frog. Whereas outline patterns emphasised the shape of the frog by following the contours of the head and along where the ilia form the lateral sides of the pelvic girdle. Due to the fragility of the gelatine models, the outer ring could not be placed precisely on the contour. Therefore, the placement could be interpreted as enhancing either the inner contour or the outline.

All models were made from a mixture of water, flavourless gelatine (Megamaxi, Ecuador), green food colouring (Megamaxi, Ecuador) and cornstarch (Megamaxi, Ecuador). Gelatine formed the main structure of the model, food colouring was used to approximate the green colour of a generic glass frog, and cornstarch was used to vary the opacity of the model (Barnett et al. 2020). For each batch of models, we mixed 52.5 g (7 × 7.5 g packets) of gelatine powder and 0.15 mL (3 drops) of green food colouring into 380 mL (180 mL cold + 200 mL boiling) of water. We then added either 0.62 g (1/4 teaspoon) or 5 g (2 teaspoons) of cornstarch to make the translucent and opaque models respectively. As each mould held ~2.5 mL of the gelatine mixture, this recipe created ~150 models.

To create the opaque patterning, we added strips of modelling clay to our gelatine models. As the bones of many glass frogs are light green (Guayasamin et al. 2020), we first dyed 32 g of white modelling clay (Doricolor, Superpaco, Ecuador) light green using 0.7 mL (14 drops) of food colouring (Megamaxi, Ecuador). The clay was then forced through a clay extruder to form strips (56 mm × 4 mm × 1 mm). The clay strips were either bent into a U‐shape or folded in half into an I‐shaped block to create two patterns of equal area. Each stripe was then placed into the gelatine mould, so that the **I**‐shaped clay formed a central dorsal‐stripe and the U‐shaped clay formed a dorso‐lateral ring around the anterior and along the sides of the frog (Figure 1D–G).

The gelatine mixture was poured into custom made frog‐shaped silicone moulds (Smooth‐Sil 940, Smooth‐On Inc., Macungie, PA, USA). The moulds were ~25 mm in length, sized to match the body length of the glass frog *Espadarana prosoblepon* (Barnett et al. 2020; Figure 1A–C), and were cast from a 3D printed model of a tree frog ( *Phyllomedusa tomopterna* ) in its resting posture (DigitalLife3D, University of Massachusetts; Barnett et al. 2020). We added the clay elements to each mould while the gelatine was still liquid. The moulds were then placed into a refrigerator and allowed to set for a minimum of 20 min. After setting, the models were removed from the moulds and allowed to fully solidify in the refrigerator overnight. Models were stored in the refrigerator for no longer than 2 days before being placed out for the field predation experiment. Refrigeration resulted in slight desiccation, which allowed the models to reabsorb ambient forest humidity and more effectively adhere to the leaves.

### 2.2 Predation Experiment

The predation experiment was conducted from July 13th to July 30th, 2024, within the foothill forests of the Mashpi Reserve. We used a randomised block design with eight treatments: the four model types (OC = opaque‐centre, OE = opaque‐outline, TC = translucent‐centre and TE = translucent‐outline) placed out at two different heights (low = ~0.5 m; high = ~1.75 m).

We established a transect along the Rio Laguna and divided the area into 60 independent blocks that were separated by a minimum of 2 m. Each block contained one replicate of each of the eight treatments and the transect as a whole contained a total of 480 models (*n* = 60 per treatment). Within each block, we randomised the treatment order and placed each model at least 1 m apart. Each model was attached to the substrate by partially melting the underside with a lighter and then sticking it, at the desired height, to the upper surface of dry, living, leaves. We ensured that each model was stuck firmly and would not fall easily without provocation by repeatedly shaking the host leaf. We repeated the experiment in three independent *rounds* in which we set out a new set of models, in the same *blocks* and along the same *transect*, but in different locations. In total, we used 1440 models (180 per treatment).

After placement, each model was monitored for evidence of attempted predation every 24‐h over a 72‐h period. Models were recorded as attacked when we observed significant marks in the gelatine that were either clearly indicative of birds (i.e., beak marks) or which could not realistically be attributed to any other physical disturbance (i.e., falling debris). Marks that were very small, or were caused by invertebrates that would not attack a live frog, were not considered as predation events. If a model had been attacked, or if it was heavily damaged, it was removed from the transect and was not replaced.

We analysed the rate of predation using a mixed‐effects Cox model survival analysis from R package *coxme* v.2.2‐22 (Therneau 2024) in R v.4.5.0 (R Core Team 2025). We included the fixed effects of *model type* (OC, OE, TC, & TE) and *height* (low & high), and the random effects of both *block* and *round*. As we had predicted the effect of height to differ according to model opacity and patterning, we fit both an interactive model (*model type* * *height*) and an additive model (*model type* + *height*) to our data. We then compared how well each model fit our data using the Akaike Information Criterion (AIC, function *AIC*) and an ANOVA test (function *anova*) from base R v.4.5.0. We checked model assumptions using the function *cox.zph* from R package *survival* v.3.8‐3 (Therneau 2024) and the significance of the main effects using the function *Anova* from R package *car* v.3.1.3 (Fox and Weisberg 2019). Where there was a main effect of *model type*, we then performed pairwise contrasts, using R package *multcomp* v.1.4.28 (Hothorn et al. 2008) and calculated relative risk of predation as hazard ratios (HR). To examine: (1) how *opacity* affected predation risk while controlling for *pattern* (i.e., OC vs. TC & OE vs. TE) and (2) how *pattern* affected predation risk while keeping *opacity* constant (i.e., OC vs. OE & TC vs. TE). We repeated these analyses twice: firstly, using a conservative estimate of bird predation where only clearly identified bird attacks were included as true events and all other missing or damaged models were counted as censored values, and secondly using a broad interpretation of predation that included all significantly damaged and missing models as attacked.

### 2.3 Photography and Visual Modelling

In order to understand the mechanisms underlying any differences in predation rate among the different experimental treatments we quantified how well each component of our model frogs matched the leaf background using visual modelling from calibrated photographs. Each model was placed onto a green leaf, as presented in the predation experiment, and was photographed using a UV sensitive, full spectrum, quartz‐converted Canon EOS 7D camera (Canon Inc., Tokyo, Japan) with a Nikkor EL 80 mm lens (Nikon Corp., Tokyo, Japan). We selected 10 replicates of each of the four model types (OC, OE, TC, & TE) and 10 green leaves from the field site. Each of the 10 replicate models was randomly paired with a different leaf and then photographed twice: once in the human visible spectrum (VIS: 400–700 nm) with the lens covered by a Baader UV‐IR blocking filter and once in the ultraviolet (UV: 300–400 nm) with the lens fitted with a Baader UV pass filter (Baader Planetarium GmbH, Mammendorf, Germany). All photographs were taken in natural daylight, were stored in RAW format, and included a 10% and a 77% Spectralon diffuse reflectance standard (Labsphere Inc., North Sutton, NH, USA). The VIS and UV photographs of one OE model could not be aligned correctly and so this replicate was excluded from the dataset.

We used the MICA Toolbox v.2.3 (Troscianko and Stevens 2015) in ImageJ v.1.54p (Schneider et al. 2012) to analyse our images. Each VIS–UV photograph pair was first converted into a linearised multispectral image using the 10% and 77% reflectance standards. We modelled the tetrachromatic, UV‐sensitive, visual system of the Eurasian blue tit ( *Cyanistes caeruleus* ; cone peak sensitivities (λ_max_): LWS = 573 nm, MWS = 508 nm, SWS = 413 nm, UVS = 372 nm and Double = 565 nm (Hart et al. 2000)) as a representative of many different UV‐sensitive bird species (Hart 2001; Hart et al. 2000; Ödeen and Håstad 2013) including potential predators of glass frogs such as motmots (Momotidae) and trogons (Trogonidae) (Dell'Aglio et al. 2018). We used the four single cones (LWS, MWS, SWS, & UVS) to calculate chromatic contrast (ΔS; hue) and the response of the double cone (D) to calculate achromatic contrast (ΔL; brightness). Our model used Weber fractions of 0.05 and, as the canopy was often broken at our field site, we modelled colour perception under D65 natural daylight.

We selected four regions of interest (ROIs) from within each photograph. (1) The base green colour of the model (*base*) to assess how gelatine opacity affected background colour matching. (2) The colour of the opaque clay patterning (*pattern*) to examine the saliency of the striped pattern and how the opacity of overlying gelatine affected the appearance of the stripes. (3) The whole model (*whole*, capturing the patterning and mean colour of the frog) to test how salient the models would be at distances where the pattern could not be resolved, and to extract pattern information for analysing background pattern matching. (4) A patch of the leaf substrate similar in size, and adjacent, to the model (*leaf*) to which we could compare the *base*, *pattern* and *whole* model ROIs.

We then used the receptor noise‐limited visual discrimination model (Vorobyev and Osorio 1998) to calculate chromatic (ΔS) and achromatic (ΔL) contrast between pairs of ROIs. To estimate how well the colour of each frog ROI matched the colour of the background ROI (i.e., background matching), we compared the *base*, *pattern* and *whole* (mean) colours to the *leaf* colour. Then, as contrast within a pattern (i.e., internal contrast) may affect target detectability we also compared the *base* and *pattern* colours of each model type. Chromatic and achromatic contrast were calculated in a manner equivalent to ‘just noticeable differences’ (JNDs), where a value < 1 suggests two colours cannot be discerned, values 1–3 are difficult to distinguish under natural lighting conditions, and values > 3 are increasingly dissimilar. We use this scale as a guide for estimating relative ease of discovery, but highlight that under natural conditions, where backgrounds and lighting may be highly heterogeneous, it is often not possible to set strict thresholds between ‘cryptic’ and ‘conspicuous’.

We were also interested in how well the patterns of our different model types matched the pattern of the leaf background. We quantified model and leaf patterning using Fast Fourier bandpass filters (i.e., granularity analysis; Stoddard and Stevens 2010; Troscianko and Stevens 2015). We used the ROIs covering the whole frog (*whole*) and the adjacent patch of leaf (*leaf*) and computed pattern energy (the standard deviation of pixel values within the achromatic channel of the visual model) at filter wavelengths ranging from 0.5 mm to approximately the width of the frog: 0.5, 1, 2, 4, 8 and 16 mm (22, 44, 88, 176, 352, 704 pixels). For each frog model, we recorded the wavelength with the highest energy (*peak spatial frequency* representing the dominant pattern size), and we calculated pattern contrast as the area between the pattern energy curves of each frog‐leaf pair using the *integrate* function from base R v.4.5.0.

We analysed background matching (ΔS and ΔL), internal contrast (ΔS and ΔL) and pattern contrast (area between pattern energy curves) using a series of generalised linear mixed effects models from R packages *lme4* v.1.1–37 (background matching and internal contrast; Bates et al. 2015) and *glmmTMB* v.1.1.11 (pattern contrast; Brooks et al. 2017; McGillycuddy et al. 2025) in R v.4.5.0. We included the fixed effect of *model type* and included *leaf ID* (background matching) or *photo ID* (internal contrast and pattern contrast) as a random factor. We checked model fit using R package *DHARMa* v.0.4.7 (Hartig 2025), the significance of the main effects using the *Anova* function from R package *car* v.3.1‐3 (Fox and Weisberg 2019), and conducted pairwise contrasts using R package *multcomp* v.1.4‐28 (Hothorn et al. 2008). We removed four outliers from the *pattern* ΔL and *whole* ΔL models to improve model fit; this did not qualitatively affect the results. We compared peak spatial frequency among model types with a Kruskal Wallis test (function *kruskal.test*) from base R v.4.5.0, and we conducted pairwise contrasts using a Dunn's test (function *dunn.test*) from R package *dunn.test* v.1.3.6 (Dinno 2024). For all analyses, pairwise contrasts were performed to assess the effect of *pattern* (i.e., centre vs. outline for both opaque and translucent: OC vs. OE & TC vs. TE) and *opacity* (i.e., opaque vs. translucent for both centre and outline patterns: OC vs. TC & OE vs. TE).

## 3 Results

### 3.1 Predation Experiment

**FIGURE 2:**
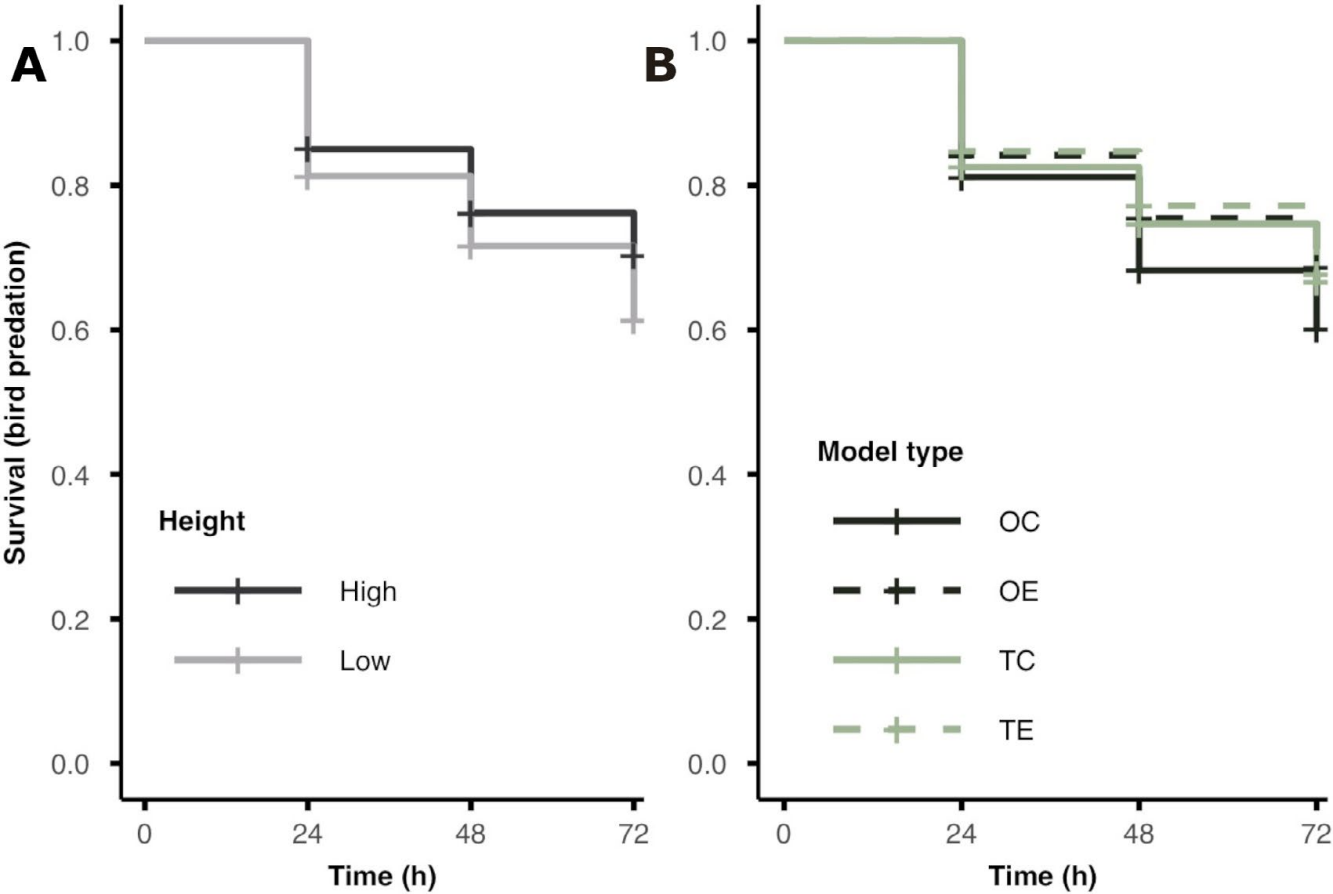
Results from the field predation study. Survival curves for the main effects of (A) perch height (black = high, grey = low) and (B) model type (colour: Black = opaque, green = translucent; line type: Solid = centre, dashed = outline | OC = opaque‐centre, OE = opaque‐outline, TC = translucent‐centre, TE = translucent‐outline) when analysing confirmed bird attacks (the results did not qualitatively differ when including all potential predation events). (A) Models on lower perches had higher risk of predation regardless of their opacity or pattern (low>high). (B) There was no effect of opacity on predation risk (OC = TC | OE = TE) and no effect of pattern on the translucent models (TC = TE) but opaque‐centre patterns had higher predation risk than opaque‐outline models (OC>OE).

When analysing the predation experiment, we were interested in the effects of both the colour pattern (*model type*: OC = opaque‐centre, OE = opaque‐outline, TC = translucent‐centre, & TE = translucent‐outline) and the height at which the models were placed (*height*: low & high; Figure 2).

We first analysed only the confirmed bird predation events (34% attack rate: 489/1440) and found that including the interaction between *model type* and *height* did not significantly improve model fit (*χ* ^2^ = 1.48, df = 3, *p* = 0.687; AIC: interactive model = 6754, additive model = 6750). We therefore interpreted the main effects of *model type* and *height* independently. There was a significant effect of perch *height*, with frogs placed on lower perches being attacked significantly more frequently than those higher up (*χ* ^2^ = 12.43, df = 1, *p* < 0.001, hazard ratio (HR) = 1.38; Figure 2A). We also found a significant main effect of *model type* (*χ* ^2^ = 8.39, df = 1, *p* = 0.039; Figure 2B) and so performed pairwise contrasts to examine how model *opacity* and *patterning* affected survival. Pairwise contrasts revealed that there was no effect of *opacity*, with no significant difference in predation risk between opaque and translucent versions of the same pattern (centre, OC‐TC: HR = 0.81, *z* = 1.72, *p* = 0.257; outline, OE‐TE: HR = 0.98, *z* = −0.14, *p* = 0.999). However, when considering the effect of *pattern*, while keeping *opacity* the same, we found that there was no difference in predation risk between the translucent models (TC vs. TE: HR = 1.09, *z* = 0.65, *p* = 0.891), but for opaque models, those with centre markings had greater predation risk than those with outline markings (OC vs. OE: HR = 0.73, *z* = 2.52, *p* = 0.042).

We found similar results when we combined both confirmed bird attacks and missing models as predation events (37% attack rate: 531/1440). The interaction between *model type* and *height* did not significantly improve model fit (*χ* ^2^ = 0.96, df = 3, *p* = 0.811; AIC: interactive model = 7356, additive model = 7351). There was a significant main effect of *height*, independent of *model type*, with lower perches experiencing greater predation risk than higher perches (*χ* ^2^ = 8.43, df = 1, *p* = 0.004; HR = 1.29). However, the main effect of *model type* was marginally non‐significant (*χ* ^2^ = 6.65, df = 3, *p* = 0.084). As this effect was marginal, we again conducted pairwise contrasts to examine the effect of *opacity* and *patterning*. There was no significant effect of *opacity* for either centre or outline patterns (centre, OC vs. TC: HR = 0.84, *z* = 1.49, *p* = 0.380; outline, OE vs. TE: HR = 0.96, *z* = −0.34, *p* = 0.982). When considering the effect of *patterning*, there was no significant difference between translucent models with centre and outline patterns (translucent, TC vs. TE: HR = 1.07, *z* = 0.52, *p* = 0.942), whereas there was a marginally non‐significant trend for opaque models with centre patterns to have higher predation risk than opaque models with outline patterns (opaque, OC vs. OE: HR = 0.75, *z* = 2.34, *p* = 0.067).

### 3.2 Background Matching—Colour Contrast

**FIGURE 3:**
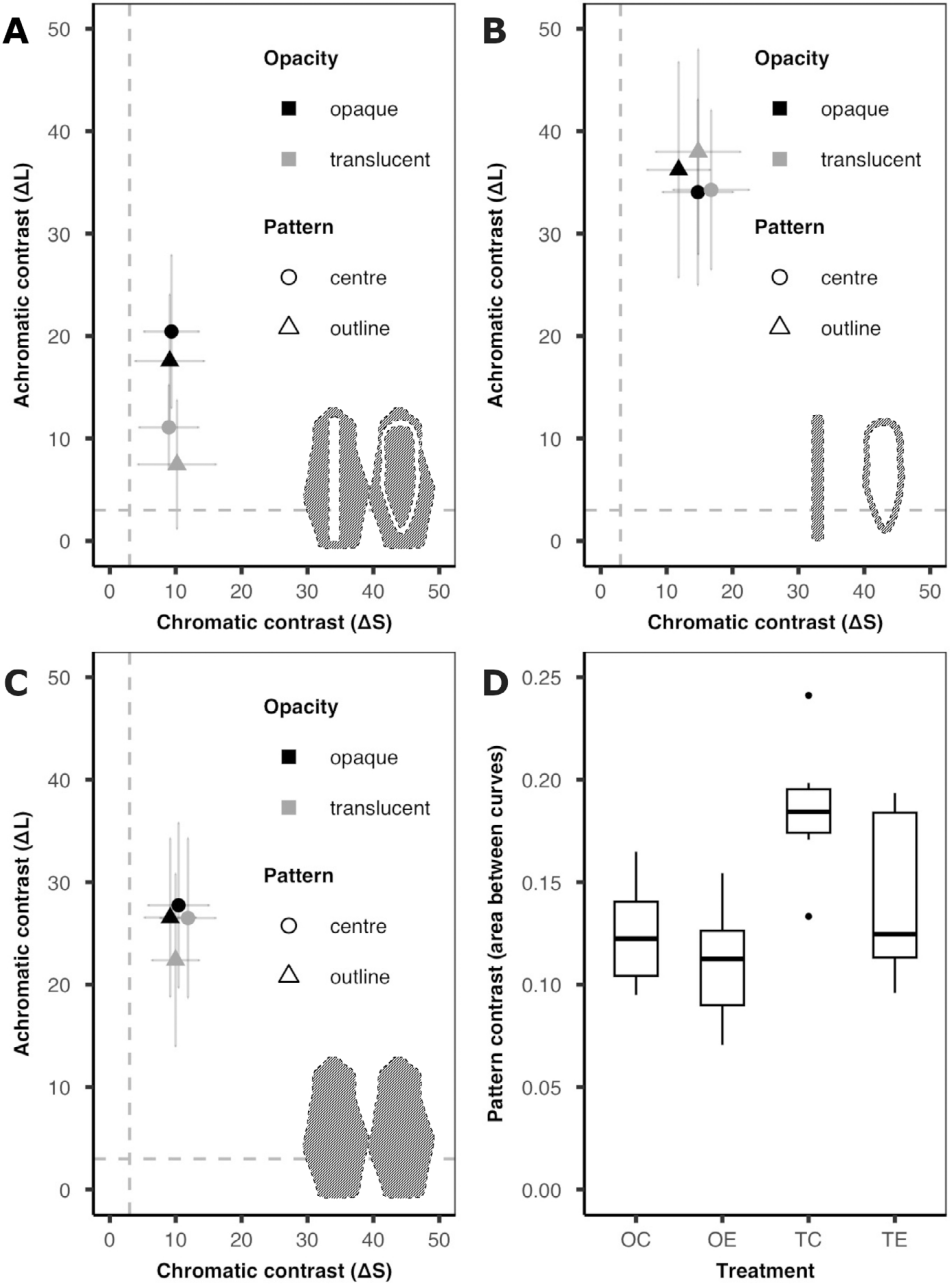
Results from the visual modelling of background matching camouflage. Colour (A–C) and pattern (D) contrast between each of the frog models used in the predation study and the green leaf background. (A–C) Colour contrast (chromatic (ΔS, x‐axis) and achromatic (ΔL, y‐axis) contrast (mean ± SD)) for the colours of each frog model type (A, base colour, B, stripe colour and C, mean colour) vs. the leaf background (opacity: Black = opaque & grey = transparent; pattern: Circle = centre & triangle = outline). Striped silhouettes indicate the area from which the ROI was selected. Dotted lines indicate the visual discrimination threshold equivalent to 3 JND. See Table 1 for summary statistics. (D) Pattern contrast area between the pattern energy curves (boxplots showing medians (crossbar), interquartile range (box) and range (whiskers)) for each of the models used in the predation study (OC = opaque‐centre, OE = opaque‐edge, TC = translucent‐centre, & TE = translucent‐outline).

To examine how the opacity of the gelatine affected how well the models matched the leaf background we compared chromatic (ΔS) and achromatic (ΔL) contrast between the *leaf* and the green *base* regions of each model (Table 1; Figure 3A). While we took a random sampling of leaves from frog habitats, we acknowledge overall leaf heterogeneity in forests is greater than our sampling. We found no significant difference among treatments in chromatic contrast (ΔS: *χ* ^2^ = 0.84, df = 3, *p* = 0.840), but treatments did differ in achromatic contrast (ΔL: *χ* ^2^ = 43.50, df = 3, *p* < 0.001). We therefore performed pairwise tests to examine the effect of *opacity* (opaque vs. translucent) and *pattern* (centre vs. outline) on achromatic contrast. There was no significant effect of patterning, with no difference in achromatic contrast between centre and outline patterns for both the opaque (OC‐OE, ΔL: *z* = 1.28, *p* = 0.514) and the translucent (TC‐TE, ΔL: *z* = 1.66, *p* = 0.286) models. However, *opacity* did affect achromatic contrast, with opaque models being more distinct from the background than were translucent models (OC‐TC, ΔL: *z* = 4.28, *p* < 0.001; OE‐TE, ΔL: *z* = 4.47, *p* < 0.001). Taken together, the green base colour of the translucent models was a closer match than the leaf background than was the base colour of opaque models, regardless of the patterning.

**TABLE 1:** Summary statistics (mean ± standard deviation) for chromatic (ΔS) and achromatic (ΔL) contrast between the base, pattern and mean colours of each model type (OC = opaque‐centre, OE = opaque‐outline, TC = translucent‐centre, & TE = translucent‐outline) versus the leaf background (background matching) and between the base of pattern colours of each model (internal contrast). See Figure 2A–C (background matching) and Figure 3A (internal contrast) for plotted data.

Model type		OC	OE	TC	TE
*Background matching*					
Base	ΔS	9.35 ± 4.15	9.09 ± 5.20	8.96 ± 4.49	10.20 ± 5.90
	ΔL	20.44 ± 7.47	17.57 ± 6.47	11.09 ± 4.12	7.46 ± 6.28
Pattern	ΔS	14.74 ± 5.33	11.81 ± 4.77	16.75 ± 5.74	14.79 ± 6.40
	ΔL	34.04 ± 9.06	36.23 ± 10.52	34.27 ± 7.78	37.98 ± 10.01
Mean	ΔS	10.43 ± 4.59	9.17 ± 3.89	11.86 ± 4.19	9.98 ± 3.56
	ΔL	27.74 ± 8.04	26.53 ± 7.72	26.49 ± 7.78	22.39 ± 8.42
*Internal contrast*					
Internal	ΔS	6.33 ± 2.13	4.85 ± 1.63	9.81 ± 4.06	7.61 ± 4.13
	ΔL	13.60 ± 2.90	18.66 ± 5.45	24.14 ± 3.96	32.17 ± 6.45

Next, to examine how pattern arrangement may affect the saliency of the opaque clay, we compared chromatic (ΔS) and achromatic (ΔL) contrast between the leaf and clay stripes of each model (Table 1; Figure 3B). We found that both chromatic and achromatic contrast differed among treatments (ΔS: *χ* ^2^ = 11.84, df = 3, *p* = 0.008; ΔL: *χ* ^2^ = 17.63, df = 3, *p* < 0.001). However, for chromatic contrast pairwise tests found no significant difference between opaque and translucent models (OC‐TC, ΔS: *z* = −1.64, *p* = 0.299; OE‐TE, ΔS: *z* = −1.90, *p* = 0.181) nor between centre and outline patterns (OC‐OE, ΔS: *z* = 1.86, *p* = 0.197; TC‐TE, ΔS: *z* = 1.59, *p* = 0.322). Whereas, for achromatic contrast there was no difference between opaque and translucent models (OC‐TC, ΔL: *z* = 0.03, *p* > 0.999; OE‐TE, ΔL: *z* = −0.67, *p* = 0.883), but centre patterns had lower achromatic contrast than did outline patterns (OC‐OE, ΔL: *z* = −2.49, *p* = 0.045; TC‐TE, ΔL: *z* = −3.33, *p* = 0.004). Therefore, although the opacity of the surrounding gelatine did not significantly affect the appearance of the clay stripes, the brightness of the outline patterns was more distinct from the leaf background than was the brightness of the centre patterns.

Finally, as the *base* and *pattern* colours would appear to blend together at greater viewing distances, we compared the mean colour of each model (*whole*) to the *leaf* background (Figure 3C). We found that both chromatic and achromatic contrast significantly differed between treatment types (ΔS: *χ* ^2^ = 20.01, df = 3, *p* < 0.001; ΔL: *χ* ^2^ = 36.30, df = 3, *p* < 0.001) and so we again performed pairwise comparisons to assess the role of *opacity* and *pattern*.

We tested the effect of *pattern* by comparing centre to outline patterns while keeping model opacity the same (i.e., OC vs. OE & TC vs. TE). The mean colours of the opaque models did not differ between patterns in either chromatic (OC‐OE, ΔS: *z* = 1.86, *p* = 0.197) or achromatic (OC‐OE, ΔL: *z* = 1.26, *p* = 0.526) contrast. Whereas the mean colour of translucent models did differ significantly between centre and outline patterns in both chromatic (TC‐TE, ΔS: *z* = 3.27, *p* = 0.004) and achromatic (TC‐TE, ΔL: *z* = 4.23, *p* < 0.001) contrast. Here, models with centre patterns were more distinct from the leaf in both instances. Next, to assess the role of *opacity*, we compared opaque and translucent models which shared the same pattern (i.e., OC vs. TC & OE vs. TE). For models with centre patterns, we found opaque models had marginally lower chromatic contrast than translucent models (OC‐TC, ΔS: *z* = −2.48, *p* = 0.047) but there was no significant difference in achromatic contrast (OC‐TC, ΔL: *z* = 1.44, *p* = 0.409). We found the opposite for models with outline patterns. There was no significant difference in chromatic contrast between opaque and translucent models (OE‐TE, ΔS: *z* = −1.10, *p* = 0.632), but opaque models had higher achromatic contrast than did translucent models (OE‐TE, ΔL: *z* = 4.20, *p* < 0.001).

These results suggest a more complex relationship between *opacity* and *pattern* for the mean colours of our models. For opaque models, pattern had no effect on background matching, whereas for translucent models, centre patterns were more distinct from the leaf background than were outline patterns. When considering the effect of *pattern*, we found that for centre patterns, translucent models had the higher chromatic contrast but there was no difference in achromatic contrast. However, for outline patterns, opaque models had higher achromatic contrast than translucent models, with there being no difference in chromatic contrast.

### 3.3 Background Matching—Pattern Contrast

To examine how closely the patterns of our frog models matched the background we calculated pattern contrast (the area between pattern energy curves measured across 0.5–16 mm) between each model and the leaf background (Figure 3D). We found that pattern contrast significantly differed between treatments (*χ* ^2^ = 38.15, df = 3, *p* < 0.001), and so we conducted pairwise tests to examine the effect of both *opacity* (opaque vs. translucent while keeping pattern the same) and *pattern* (centre vs. outline while controlling opacity).

When considering *opacity*, we found that for central patterns, opaque models were a closer match to the background pattern than translucent models (OC‐TC: *z* = −4.92, *p* < 0.001). Whereas for outline patterns, although there was a trend for opaque patterns to more closely match the background, this effect was marginally non‐significant (OE‐TE: *z* = −2.19, *p* = 0.098). When analysing the effect of *pattern*, we found that there was no difference in pattern contrast between opaque centre and opaque outline treatments (OC‐OE: *z* = 0.87, *p* = 0.776). However, for translucent models, centre patterns were significantly more distinct from the leaf than outline patterns (TC‐TE: *z* = 3.56, *p* = 0.002).

Taken together these results indicate that the effect of clay patterning differed depending on the opacity of the surrounding gelatine, with pattern contrast being higher for translucent models than opaque models. Moreover, we also found that the opacity of the surrounding gelatine altered the effect of pattern, with centre patterns being more distinct from the leaf background than were outline patterns; but this effect was only evident for the translucent models.

### 3.4 Model Patterning—Internal Contrast

**FIGURE 4:**
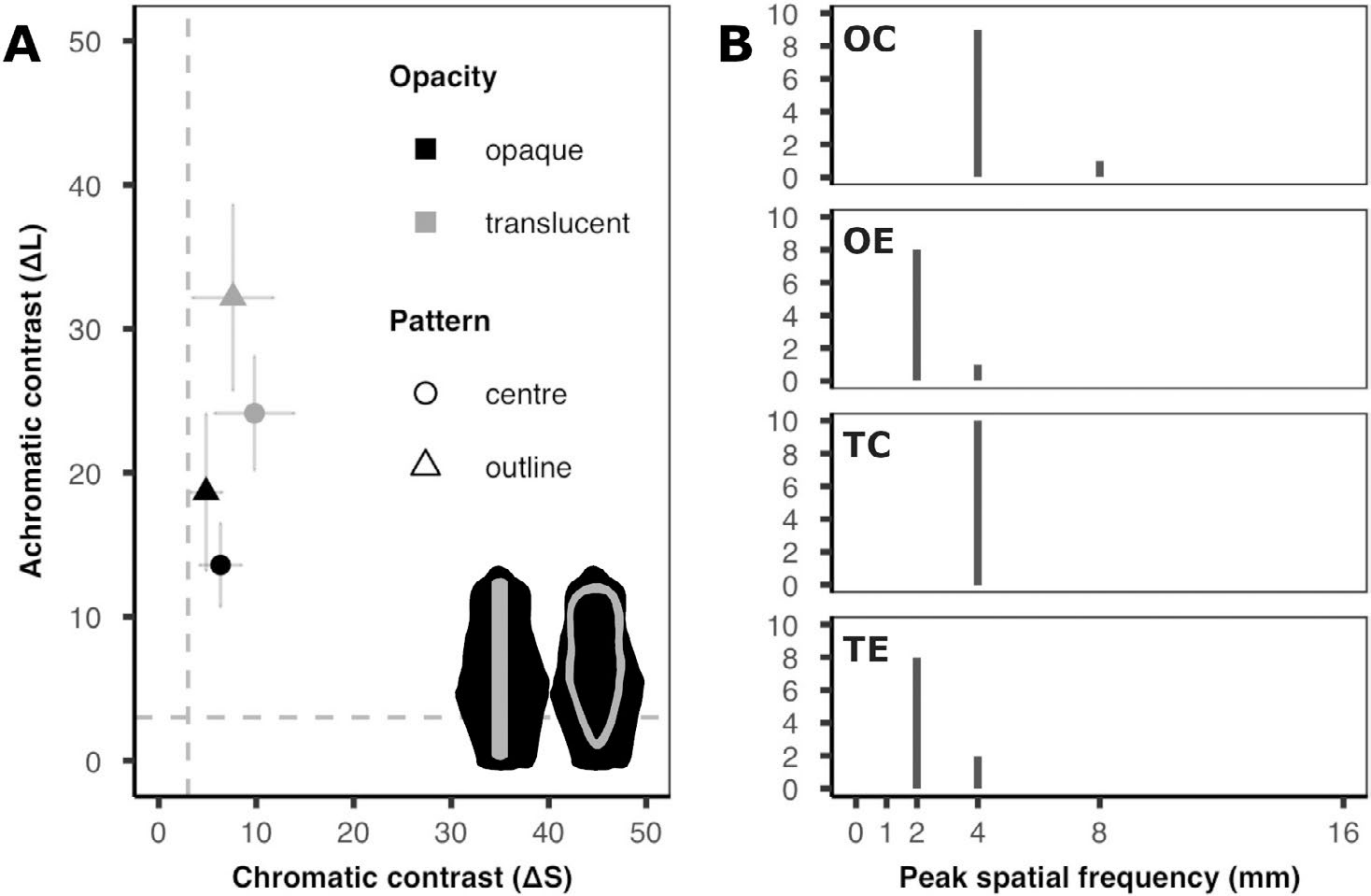
Results from the visual modelling of model colour pattern characteristics. (A) Internal colour contrast (chromatic (ΔS) and achromatic (ΔL) contrast (mean ± SD)) between the base and stripe colours for each treatment (opacity: Black = opaque & grey = transparent; pattern: Circle = centre & triangle = outline). Model silhouettes show the regions from which the ROIs were selected, for each model, internal contrast was calculated between the black and grey regions. Dotted lines indicate the visual discrimination threshold equivalent to 3 JND. Chromatic contrast is significantly greater in translucent models than in opaque models. Achromatic contrast was higher in translucent models than in opaque models, and higher for edge patterns than for centre patterns. See Table 1 for summary statistics. (B) Peak spatial frequency (histograms of the dominant pattern size in mm) for each model treatment (*n* = 10 per treatment; OC = opaque‐centre, OE = opaque‐outline, TC = translucent‐centre. TE = translucent‐outline). Pattern energy was calculated at 0.5, 1, 2, 4, 8 and 16 mm. The two treatments with outer patterns (OE & TE = 2 mm thick stripes) have significantly thinner stripes than those with centre patterns (OC & TC = 4 mm thick stripes).

As target saliency often depends on the contrast between pattern components, we next quantified the contrast within the pattern of each model (i.e., the base green vs. the opaque stripes, Figure 4). We found that internal contrast differed between treatments for both chromatic and achromatic contrast (ΔS: *χ* ^2^ = 13.42, df = 3, *p* = 0.004; ΔL: *χ* ^2^ = 126.88, df = 3, *p* < 0.001). We therefore performed pairwise tests to examine the role of *opacity* and *pattern*. When considering chromatic contrast, we found no difference between centre and outline patterns for both opaque (OC‐OE: *z* = 1.04, *p* = 0.675) and translucent (TC‐TE: *z* = 1.61, *p* = 0.314) models. However, although there was no effect of *opacity* on outline patterns (OE‐TE: *z* = −1.94, *p* = 0.167), opaque centre patterns had marginally lower chromatic contrast than translucent centre patterns (OC‐TC: *z* = −2.54, *p* = 0.039). Conversely, when analysing achromatic contrast, we found that opaque models had lower contrast than translucent models regardless of *pattern* (OC‐TC: *z* = −6.15, *p* < 0.001; OE‐TE: *z* = −7.45, *p* < 0.001) and that centre patterns had lower contrast than outline patterns irrespective of *opacity* (OC‐OE: *z* = −3.04, *p* = 0.008; TC‐TE: *z* = −4.69, *p* < 0.001). Taken together our data reveal that the major differences in internal contrast are found in brightness (i.e., achromatic contrast) with translucent models having higher contrast than opaque models and centre patterns having high contrast than outline patterns.

### 3.5 Model Patterning—Peak Spatial Frequency

We also compared the peak spatial frequency (i.e., the dominance pattern size) of each model type. We found that pattern size significantly differed between treatments (*χ* ^2^ = 27.59, df = 3, *p* < 0.001). We therefore performed pairwise contrasts to examine how peak spatial frequency differed according to model *opacity* and *pattern*. We found there to be no significant difference between opaque and transparent versions of the same pattern (OC‐TC: *z* = 0.26, *p* = 1.00; OE‐TE: *z* = −0.37, *p* = 1.00). However, central patterns had thicker striping than did outline patterns for both the opaque (OC‐OE: *z* = 3.97, *p* < 0.001) and transparent (TC‐TE: *z* = 3.44, *p* = 0.001) models.

## 4 Discussion

To be transparent appears to represent the quintessential, idealised form of concealment (Cuthill 2019; Barnett et al. 2025). However, although there are many remarkable examples of partially see‐through organisms, true transparency is rare and many bodily structures may be too thick or complex to allow for uninterrupted light transmission (Johnsen 2001; Bagge 2019; Barnett et al. 2025). Here we examined how obligate opaque structures contained within the bodies of translucent glass frogs (i.e., the internal organs) may affect the efficacy of camouflage and asked whether greater translucency could disguise salient opaque patches through disruptive camouflage.

We predicted that translucent patches would help conceal adjacent opaque areas by facilitating more effective disruptive camouflage (Stevens and Merilaita 2008; Merilaita et al. 2017; Barnett et al. 2025). Correspondingly, we also predicted that opaque patterns which highlighted the characteristic shape of the frog would be more easily recognised and experience greater predation risk, compared to patterns where the outline was translucent. Considering both the field and modelling data together, we indeed found that areas with higher levels of light transmission were a better match to the brightness of the immediate background (Figures 1A,B and 3A). In a previous study, using similar models but without the opaque patterning, we found that such differences in opacity resulted in translucent models having a lower risk of predation (Barnett et al. 2020). Here, however, when models included salient opaque patterns, we found no evidence that greater translucency affected the frequency of predation. Our data therefore suggest that the presence of salient opaque regions may reduce the camouflaging effects otherwise conferred by the presence of transparent or translucent patches.

Previous work, using artificial moth‐like targets, does suggest that small transparent patches on an otherwise opaque surface can facilitate camouflage through both edge and surface disruption (Stevens and Merilaita 2008; Arias et al. 2019, 2021; Costello et al. 2020). We did not replicate this finding and instead found that predation risk was not primarily driven by the degree of translucency. This finding may result from the high luminance contrast produced by the opaque patterns used in our study, which were more contrasting against the leaf background than either the translucent or opaque base colours. Whereas in previous work opaque surfaces have matched the luminance or colour of the background (Arias et al. 2019, 2021; Costello et al. 2020), our opaque patterns were instead more reminiscent of bones (e.g., the vertebrae and the ilia), which can be visible through the skin of some glass frogs, and the viscera (e.g., the gut, pericardium, or peritoneum) which in glass frogs frequently appears white due to the presence of iridophores (Guayasamin et al. 2020; Taboada et al. 2022; Figure 1B). Disruptive camouflage has been suggested to function even with high contrast colours not found in the immediate background, but efficacy is highest when disruptive contrast is produced using colours found within the environment (Stevens and Merilaita 2008; Cuthill 2019). As such, the white viscera may be too salient of a feature to conceal through differential blending, regardless of their placement within the body.

We did find a marginally significant trend for centre patterns to have greater predation risk than outline patterns, but only for the opaque models and only when restricting our analysis to the confirmed bird attacks. This finding is at odds with our initial prediction that outline patterns would be more easily recognised and so would be attacked more frequently than centre patterns. However, as all patterns used in our study contained the same area of opaque material, this result may instead be a product of differences in the distances at which the two patterns could be resolved (Barnett et al. 2017; Postema et al. 2023). The thicker central bar was likely more clearly visible from a greater distance than the two, thinner outer stripes which would appear to blend with the surrounding green gelatine to form a more cryptic average colour at a shorter range (Barnett et al. 2017).

Our data therefore suggest that translucency may mitigate some of the detectability costs associated with the presence of opaque patterns. However, the high saliency of certain opaque structures within the body might preclude glass frogs from being fully transparent such that greater transparency of the dorsal skin may paradoxically be detrimental for effective concealment. If the dorsal skin were to be as transparent as the ventral skin, the highly contrasting internal organs could negate the benefits of transparent camouflage. Opaque structures themselves may therefore benefit from being pigmented (i.e., the bones of many glass frogs are green (Guayasamin et al. 2020)), but when surrounded by transparent regions, distinct and salient contrast boundaries may remain. Indeed, we found that these internal contrast boundaries may be even more distinct when opaque patterns are surrounded by transparent regions or covered by a thinner layer of translucent material. As an alternative, a gradient of pigmentation, from the more translucent edge to the more opaque centre of the body, may conceal these contrast boundaries and facilitate ‘edge diffusion’. Here translucency may improve concealment even if the opaque, pigmented colour does not fully match that of the background (Barnett et al. 2020, 2025; Webster et al. 2015). Edge diffusion as a form, or component, of camouflage has not yet been studied in detail, and more research is needed to understand when and where edge diffusion may function (Barnett et al. 2025).

We also hypothesised that glass frog translucency may help to disguise the shape of the silhouette, and so predicted that efficacy of translucency camouflage could be influenced by the perching height. Many glass frogs are predominantly found living on leaves high off the ground (JY, JBB pers. obvs.) and so may be detected both by reflected light from the frog's dorsum (i.e., predators viewing the frog from above) and by the silhouette they cast when backlit (i.e., predators viewing from below/behind the leaf on which the frog is perched). We therefore predicted that predation rates would be highest where predators are able to approach from any angle, where both the frog and its silhouette would be visible, and where the silhouette cast would highlight the outline of the frog (i.e., predation would be highest on opaque frogs with outline patterns which were perched higher off the ground). Instead, we found that frogs on lower perches were attacked more frequently regardless of pattern or opacity. As the silhouette of lower placed models would not be visible to the majority of predators, this suggests that silhouette driven predation was not a major factor in our experiment. Rather, lower models may have simply been more accessible to a larger number of predators including terrestrial (e.g., guans) as well as arboreal (e.g., motmots) foraging species. We were therefore unable to fully examine the potential role of transparency in silhouette concealment and call for further research into whether silhouettes may be an important factor in the evolution of partial or imperfect transparency particularly at higher resting heights. Nonetheless, differential predation risk based on perch height is an interesting finding which may affect frog behaviour, such as site selection during rest or parental care, and so warrants further research.

Taken together we found that although greater translucency did result in more effective background luminance matching, increasing translucency did not reduce predation rates on frogs with conspicuous opaque structures within their bodies. Instead, background matching pigmentation may be required to block the view of these opaque structures. Indeed, green pigmentation is universally found on the dorsal surfaces of glass frogs, and many species also display spots, netting and other patterned elements which vary in size, pigmented colour and in translucency (Guayasamin et al. 2020; Guillory et al. In Review). Future studies are therefore needed to understand how these pattern components may interact with opaque and translucent body regions to alter the efficacy of camouflage both with reflected and transmitted light.

Our results contribute to a growing body of research into how, rather than being a simple story of background matching, transparency may instead facilitate many different forms of concealment (Barnett et al. 2025). Although transparency may appear to be the ultimate form of camouflage, we find that greater transparency does not always compensate for the presence of salient opaque structures which may remain due to physiological limitations in tissue composition (Johnsen 2001; Bagge 2019; Cuthill 2019; Barnett et al. 2025). For glass frogs, translucency may then retain many of the benefits of transparency (i.e., dynamic luminance matching) while utilising pigments to minimise the saliency of opaque tissues and organs (Barnett et al. 2020; Taboada et al. 2022). Further work is needed to examine the physiological limits of transparency and how partial transparency may facilitate different anti‐predatory strategies including various forms of camouflage but also the potential role of aposematic (warning) or communicative signals.

## Author Contributions

**Justin Yeager:** conceptualization (equal), data curation (equal), funding acquisition (lead), investigation (equal), methodology (equal), project administration (equal), resources (equal), supervision (equal), writing – original draft (equal), writing – review and editing (equal). **Abigail Robison:** data curation (equal), investigation (equal), writing – review and editing (equal). **Cordon D. Wade:** data curation (equal), investigation (equal), writing – review and editing (equal). **James B. Barnett:** conceptualization (equal), data curation (equal), formal analysis (lead), investigation (equal), methodology (equal), project administration (equal), supervision (equal), validation (equal), visualization (equal), writing – original draft (equal), writing – review and editing (equal).

## Funding

This work was supported by the Universidad de las Américas, Quito, Ecuador (UDLA) grant (483.A.XIV.24).

## Ethics Statement

Experiments were approved by the Ministerio del Ambiente, Ecuador (Permits: MAATE‐ARSFC‐2022‐2694 and MAATE‐ARSFC‐2024‐0029).

## Conflicts of Interest

The authors declare no conflicts of interest.

## Data Availability

Data is available from Dryad: https://doi.org/10.5061/dryad.xd2547dw6.
